# Effects of COVID-19 on the contrast sensitivity

**DOI:** 10.1590/1414-431X2025e14517

**Published:** 2025-05-09

**Authors:** G.M. Silva, J.J.S Souto, T. Fernandes, G.S. Souza, M.J.O. de Andrade, N.A. dos Santos

**Affiliations:** 1 Laboratório de Percepção, Neurociências e Comportamento, Universidade Federal da Paraíba, João Pessoa, PB, Brasil; 2 Laboratório de Medicina Tropical, Universidade Federal do Pará, Belém, PA, Brasil; 3 Laboratório de Neurociências, Cronobiologia e Psicologia do Sono, Universidade do Estado de Minas Gerais, Divinópolis, MG, Brasil

**Keywords:** COVID-19, Coronavirus, Contrast sensitivity function, Psychophysics

## Abstract

There are significant gaps in understanding the extent of the damage caused by COVID-19, with few publications examining its link to contrast sensitivity function (CSF). The aim of the present study was to evaluate CSF at low, medium, and high spatial frequencies in individuals with and without a history of COVID-19. Thirty adults, both male and female, aged between 18 and 49 years, participated in the study, 15 with a history of COVID-19 and 15 without. CSF was measured using Metropsis software (version 11) and vertical sine-wave gratings with spatial frequencies ranging from 0.2 to 19.8 cycles per degree (cpd). The results indicated COVID-19-related changes in CSF at spatial frequencies of 6.1 (U=36.00; P=0.003; r=-0.55), 13.2 (U=29.00; P=0.001; r=-0.61), 15.9 (U=17.00; P=0.001; r=-0.70), and 19.8 cpd (U=13.00; P=0.001; r=-0.73). The observed decrease in CSF within specific spatial frequency bands suggested that the visual system of individuals exposed to COVID-19 required higher contrast levels to detect high spatial frequencies. This psychophysical finding indicated that COVID-19 altered the functioning of the visual system and likely affected the neural mechanisms responsible for processing high spatial frequencies.

## Introduction

COVID-19, a disease caused by the SARS-CoV-2 virus, is notable for its transmissibility and systemic effects that extend beyond the respiratory tract and affect multiple bodily functions, including the central and peripheral nervous systems (1). In addition to flu-like symptoms, studies indicate that between 2 and 32% of patients diagnosed with COVID-19 may experience visual changes since the acute phase of the disease (2,3), including hyperreflective lesions at the level of ganglion cells (4), Purtscher-type retinopathy, with multiple bilateral cotton-wool spots (5), and optic nerve hyperemia in the right eye, optic nerve pallor in the left eye, arteriolar attenuation, multiple cotton-wool spots, and ill-defined areas of retinal clearing at the posterior pole in both eyes (6). Cranial nerve palsies (involving nerves III, IV, and VI) (7) and visual field deficits have also been reported, reflecting the broader neurological impact on visual functions (8).

The pathophysiology of these problems has not yet been fully clarified (9,10). However, some studies have proposed a probable pathophysiological mechanism of SARS-CoV-2 infection through the ocular surface (11,12). The main indicator is the presence of the ACE2 receptor in ocular tissue (13,14), given that the human eyeball has its own intraocular renin-angiotensin system, which is present both on the surface (cornea and conjunctival epithelial cells) and inside the eye (15).

However, a review has shown that both ocular tissue tropism and the prevalence of high viral load in this region are relatively low (9). Although there is little evidence to support the hypothesis that the ocular surface is one of the sources of virus dissemination, it is possible to establish relationships between the presence of SARS-CoV-2 receptors in ocular tissue and visual manifestations in patients affected by the disease (11).

These changes indicate that COVID-19 is a potentially neuroinvasive disease that affects the central nervous system (CNS) (16). However, the extent of the damage caused by the disease, such as the impact on visual functions, is not well known (17). The existing reports on the effects of COVID-19 on visual processing are limited and have focused on ocular manifestations and structural changes. Neural pathways and the underlying mechanisms of visual processing, such as those assessed in both achromatic and chromatic conditions, remain underexplored.

An important study on congenital zika syndrome (CZS) demonstrated reduced visual acuity, which correlated with functional deficits in motor tasks such as object tracking and reaching, but was not associated with cognitive performance (18). These findings offer key insights: a) viral infections like CZS and COVID-19 share neurological influences, as both affect the CNS via similar mechanisms; b) both conditions impact multiple systems, highlighting their interconnectedness; and c) persistent viral infections, whether due to CZS or COVID-19, may underlie long-term neurological and systemic impairments. Research on CZS serves as a valuable model for understanding fundamental aspects of viral pathogenesis, including its sensory and neurological components, which may inform our knowledge of similar mechanisms in COVID-19. While this study offered valuable insights, its focus solely on visual acuity limited the broader understanding of visual function. Such a narrow approach may overlook subtle or complex sensory deficits that contribute to overall visual impairments.

The study of basic visual functions through psychophysical methods is a noninvasive way to investigate the impact of different conditions on visual processing and the CNS, as is the case with COVID-19 (19). One of the main basic visual functions studied in the literature is the contrast sensitivity function (CSF), which refers to the ability to discriminate stimuli or visual patterns with different luminance levels (20). The use of CSF to describe changes in the CNS has grown over the years. The literature suggests that various conditions, including aging, chronic diseases, neuropsychiatric disorders, and viral infections affecting the CNS, can influence the CSF (21).

The effects of COVID-19 on CSF are still unclear, as there are few publications on the subject. One of the studies conducted a prospective case-control study with 63 cases and 128 controls in 2021-2022 using the ‘smart optometry' mobile app and the free online version of the Farnsworth-Munsell 100 hue test by X-Rite (USA). The results indicated impairments only in color vision (22).

The present research aimed to investigate the effects of COVID-19 on CSF in people with and without a history of the disease.

## Material and Methods

This study was approved by the Research Ethics Committee of the Health Sciences Center at the Federal University of Paraíba (CAAE: 46067721.3.1001.5188). Written consent was obtained from all participants prior to the start of the study.

### Participants

The study included 30 volunteers aged 18 to 49 years (mean=27.1; SD=7.10 years). We compared two groups of participants, each comprising 15 volunteers. The study group (SG) included participants with a history of a COVID-19 diagnosis. The control group (CG) was sourced from our laboratory's pre-pandemic database to ensure that these participants had no exposure to the virus.

All participants had normal or corrected-to-normal visual acuity. No participant had a history of ocular or neurological disease. Sampling was non-probabilistic (convenience sampling) and conducted through social media outreach.

### Eligibility criteria

#### Inclusion criteria

1) Age between 18 and 50 years; 2) Normal or corrected visual acuity; 3) Absence of disorders or pathologies affecting visual functions and the central nervous system; 4) COVID-19 diagnosis confirmed through laboratory criteria, such as reverse transcription polymerase chain reaction (RT-PCR) or immunological tests (rapid test or standard serology for antibody detection) with a positive result for immunoglobulin antibodies (IgM and/or IgG) or clinical-epidemiological criteria, including a history of close or household contact with an infected person.

#### Exclusion criteria

1) Current viral infections from etiological agents other than SARS-CoV-2 or other diseases affecting the respiratory tract with a negative COVID-19 test; 2) Use of illicit psychoactive substances; 3) Diagnosis of diabetes and/or hypertension; 4) Occupational exposure to organic solvents and/or heavy metals.

### Instruments

For the screening phase, the following instruments were used: a sociodemographic and clinical data questionnaire, the Mini-Mental State Examination (MMSE), the Beck Depression Inventory II (BDI-II), and the Beck Anxiety Inventory (BAI).

### Stimuli and equipment

#### Achromatic contrast sensitivity

The Metropsis software (Cambridge Research Systems, UK) was used to measure the contrast threshold. Vertical sine-wave grating stimuli, defined in Cartesian coordinates with spatial frequencies of 0.2, 0.6, 1.0, 3.1, 6.1, 8.8, 13.2, 15.9, and 19.8 cycles per degree of visual angle (cpd) were used. All stimuli were generated in grayscale, with an approximately 7.2 degrees of visual angle and presented at a distance of 150 cm on a 19-inch LG CRT video monitor with a spatial resolution of 1024×786 pixels, a temporal resolution of 100 Hz, and an average luminance of approximately 40 cd/m^2^.

### Procedure

The study was conducted in a single experimental session divided into two stages, each lasting approximately 30 min.

The first stage involved the screening process, during which participants completed the sociodemographic and clinical data questionnaire, as well as the BAI, BDI, and MMSE scales. Visual acuity was also assessed during this phase using Rasquin's “E” optotypes. Participants were positioned 6 m away from the optotype chart and asked to verbally indicate the direction of the “E” optotype opening.

The second stage consisted of measuring contrast thresholds under photopic conditions and binocularly. Participants were seated on a chair 150 cm away from the monitor displaying the stimuli.

**Figure 1:**
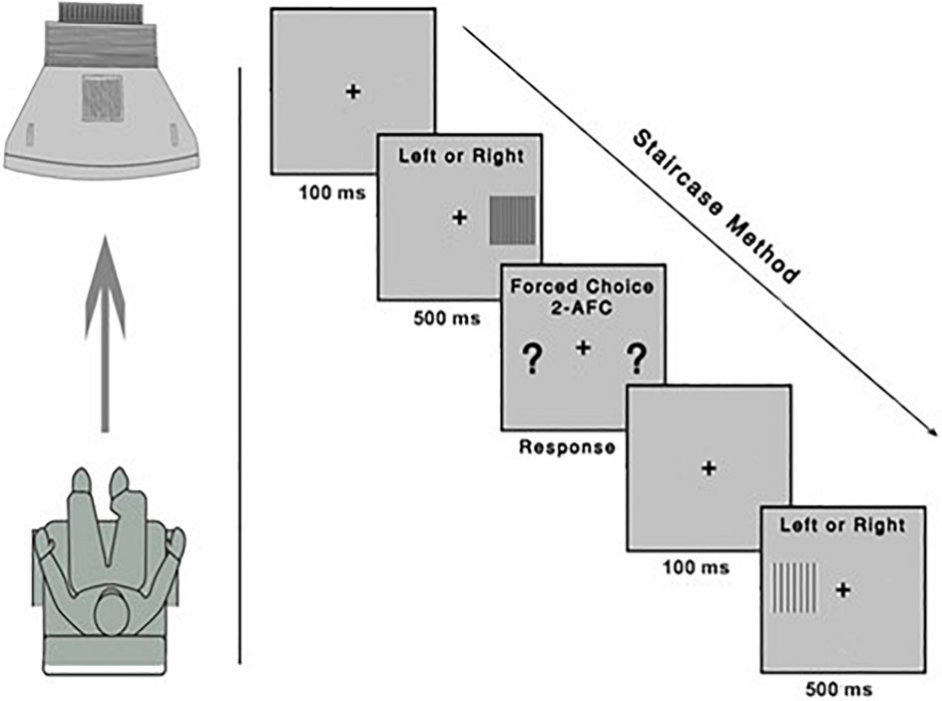
Schematic representation of the application of the test to measure the contrast threshold (adapted from Fernandes et al. 2019 (23)). The Metropsis algorithm randomizes spatial frequencies (low, medium, and high) and contrast values. 2-AFC: two-alternative forced choice.

The test uses the psychophysical two-alternative forced choice (2-AFC) method. During its application, participants indicated the side of the screen on which the stimulus appeared by pressing the corresponding button on the response box of the CB6 model (Cambridge Research Systems). The red button was pressed if the stimulus appeared on the right side, and the black button if it appeared on the left. In cases where participants were unsure of the stimulus location or could not detect it because it was below the contrast threshold, they were instructed to press any button and guess. Figure 1 provides a diagram illustrating the test conditions.

The dynamic staircase method on a logarithmic scale was used. This method dynamically adjusts stimulus contrast after each trial by decreasing by 0.7 dB following three consecutive correct responses and increasing by 1 dB after one incorrect response, converging at 79.4% accuracy on the psychometric function (23). The test began with a supra-threshold stimulus. Each stimulus was presented for 500 ms, with a 100 ms interval between presentations. The stimulus presentation time was determined based on previous studies, which found that short presentation times are sufficient for participants to provide responses at the sensory processing level (23,24). The test ended automatically after 10 reversals (maximum and minimum threshold values) for each spatial frequency in the protocol.

### Statistical analysis

IBM SPSS Statistics software version 21 (USA) was used for data analysis. To characterize the sample, descriptive analyses were performed using measures of central tendency (e.g., mean, median) and dispersion (standard deviation). The Shapiro-Wilk normality test was conducted to assess the distribution of data for spatial frequencies. Since not all data followed a normal distribution (P<0.05), non-parametric analyses were performed using the Mann-Whitney U and Kruskal-Wallis tests. Multicollinearity and variance-covariance were assessed. No outliers were identified. The Bonferroni correction was applied, and the significance value (P) was adjusted by dividing it by the number of comparisons made.

## Results

### General characteristics of the sample

Table 1 presents an overview of the participants' biosociodemographic characteristics. In general, most participants were female (60%), unmarried (73.33%), with higher education (56.33%), and a monthly income above three minimum wages (40%).

**Table 1:** Biosociodemographic characteristics of the study group and control group participants.

Characteristics	Study group(n=15)	Control group(n=15)
Age, years (SD)	28 (8.92)	26.27 (4.89)
Sex (%)		
Male	5 (16.67)	7 (23.33)
Female	10 (33.33)	8 (26.67)
Marital status (%)		
Single	13 (43.33)	10 (33.33)
Married	2 (6.67)	5 (16.67)
Level of education (%)		
Incomplete college	7 (23.33)	6 (20.00)
Graduated	8 (26.67)	9 (30.00)
Salary		
<1 minimum salary	1 (3.33)	-
1 minimum salary	1 (3.33)	2 (6.67)
1-2 minimum salaries	2 (6.67)	-
2 minimum salaries	1 (3.33)	6 (20.00)
3 minimum salaries	1 (3.33)	4 (13.33)
>3 minimum salaries	9 (30.00)	3 (10.00)

The Mann-Whitney U test did not reveal statistically significant differences between the two groups for the sociodemographic variables: gender (U=97.50; P=0.46), age (U=108.00; P=0.85), marital status (U=90.00; P=0.20), and education (U=105.00; P=0.72).

Table 2 presents the clinical data regarding COVID-19. Most participants had a diagnosis confirmed through RT-PCR (93.33%) and experienced mild symptoms of the disease (93.33%). The most frequent symptoms were: headache (60%), anosmia/hyposmia (53.33%), ageusia/hypogeusia (46.67%), and fever (46.67%). More than half of the SG participants used some medication during the period of disease symptoms (53.33%), either alone (20.00%) or in combination with at least one other medication (33.33%). The most commonly listed medications were azithromycin (46.67%) and ivermectin (20.00%). SG participants took part in the research between 24 and 341 days after their diagnosis. Only four of these participants required hospitalization. Of the 30 participants who completed the survey, only seven (23.33%) had been vaccinated against COVID-19, all of whom were from the SG.

**Table 2:** Clinical characteristics of COVID-19 of the study group participants.

Characteristics	Study group (n=15)
Test type (%)	
RT-PCR	14 (93.33)
Uninformed	1 (6.67)
Time between diagnosis and research participation, days (SD)	167.90 (94.74)
Hospitalization (%)	4 (13.33)
Severity of symptoms (%)	
Oligosymptomatic	14 (93.33)
Moderate	1 (6.67)
Symptoms (%)	
Ageusia/hypogeusia	7 (46.67)
Anosmia/hyposmia	8 (53.33)
Tiredness	6 (40.00)
Headache	9 (60.00)
Runny nose	3 (20.00)
Diarrhea	2 (13.33)
Difficulty speaking	1 (6.67)
Dyspnea	3 (20.00)
Body ache	5 (33.33)
Chest pain	1 (6.67)
Lack of appetite	1 (6.67)
Fever	7 (46.67)
Sore throat	3 (20.00)
Cough	4 (26.67)
Vertigo	1 (6.67)
Medication use (%)	8 (53.33)
Medication Type	
Azithromycin	7 (46.67)
Chloroquine	1 (6.67)
Dexamethasone	1 (6.67)
Dipyrone	1 (6.67)
Ivermectin	3 (20.00)
Prednisone	1 (6.67)
Vaccine (%)	
AstraZeneca	5 (33.33)
Janssen	1 (6.67)
Pfizer	1 (6.67)

Participants had a mean score of 8.90 (SD=7.72) in the BAI, 10.97 (SD=11.41) in the BDI, and 29.50 (SD=0.73) in the MMSE. Medians and interquartile ranges for each group are shown in Table 3. The Mann-Whitney U test did not indicate statistically significant differences between the groups for the BAI (U=100.00; P=0.60), BDI (U=97.00; P=0.52), and MMSE (U=106.50; P=0.77).

**Table 3:** Values in the Beck Anxiety Inventory (BAI), Beck Depression Inventory II (BDI), and Mini-Mental State Examination (MMSE) scales for the study group and control group.

Scales	Study group (n=15)		Control group (n=15)		
	Median	IQR	Median	IQR	P-value
BAI	7	8.5	6	4.5	0.60
BDI	8	8.5	9	1.5	0.52
MMSE	30	0.5	30	0.5	0.77

Data are reported as median and interquartile range (IQR). Mann-Whitney U test.

### Contrast sensitivity

**Figure 2:**
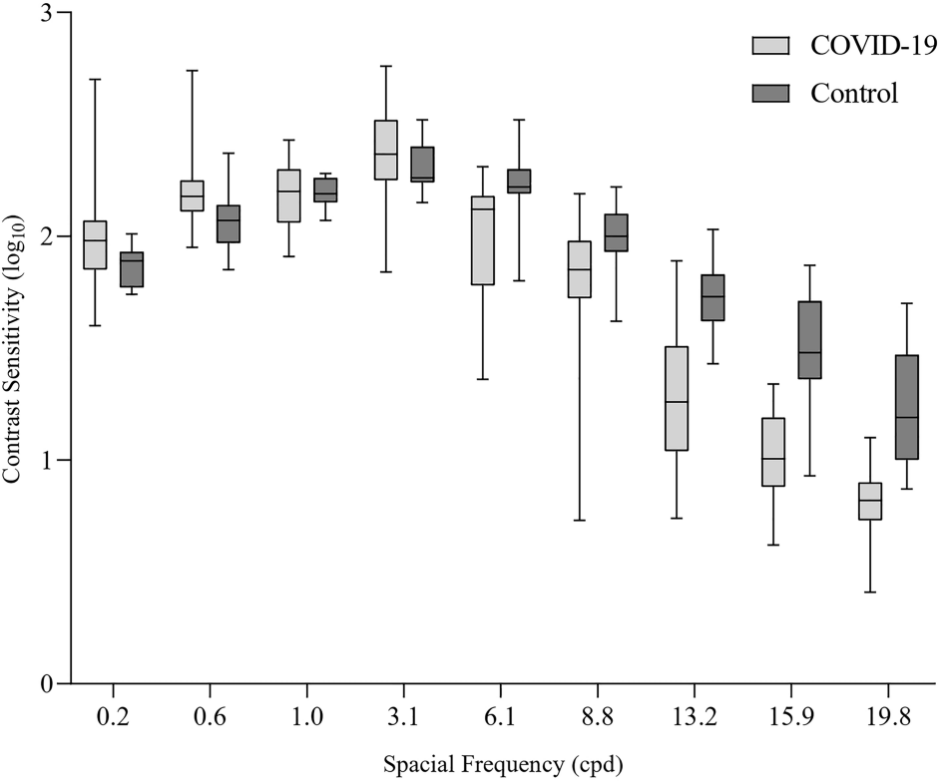
Contrast sensitivity values for the COVID-19 group and control group. Lines in boxes are median and interquartile range and whiskers represent maximum and minimum contrast sensitivity values. cpd: cycles per degree.

The results of the CSF measurements are shown in Figure 2. A Bonferroni correction was applied, and all effects were tested at the 0.025 significance level. The Mann-Whitney test showed significant differences between the two groups for the CSF of the spatial frequencies 6.1 (U=36.00; P=0.003; r=-0.55), 13.2 (U=29.00; P=0.001; r=- 0.61), 15.9 (U=17,00; P=0.001; r=-0.70), and 19.8 cpd (U=13,00; P=0.001; r=-0.73). In general, SG showed lower contrast sensitivity compared to CG for spatial frequencies above 6.1 cpd, except for 8.8 cpd. However, no statistically significant differences were identified for frequencies 0.2 (P=0.097), 0.6 (P=0.055), 1.0 (P=0.600), 3.1 (P=0.256), and 8.8 cpd (P=0.032).

In addition, medians and interquartile ranges of contrast sensitivity for each spatial frequency can be seen in Table 4.

**Table 4:** Contrast sensitivity values for the study group and control group.

Spatial frequencies (cpd)	Study group(n=15)		Control group(n=15)		
	Median	IQR	Median	IQR	P-value
0.2	92.93	28.15	78.43	13.14	0.097
0.6	149.38	25.64	117.65	22.45	0.055
1.0	160.66	43.21	153.85	19.48	0.600
3.1	225.88	85.92	181.82	37.73	0.256
6.1	138.46	48.87	166.67	23.08	0.003
8.8	74.72	26.93	100.00	19.95	0.032
13.2	16.68	12.48	53.33	13.28	0.001
15.9	10.49	4.29	30.53	14.17	0.001
19.8	6.91	1.54	15.50	9.59	0.001

Data are reported as median and interquartile range (IQR). Mann-Whitney U test. cpd: cycles per degree.

### Subgroup analysis

To estimate in detail the effects of COVID-19 on CSF, subgroup analyzes were conducted.

In the first analysis, SG participants were divided into three subgroups, according to the interval between the date of COVID-19 diagnosis and the date of study participation: ≤100 days (n=3), ≤200 days (n=3), and >200 days (n=4). Bonferroni correction was applied and all effects were tested at the 0.017 level of significance. The Kruskal-Wallis test did not show statistically significant differences between the subgroups for any of the spatial frequencies: 0.2 [X^2^ (2)=3.755; P=0.153], 0.6 [X^2^ (2)=3.027; P=0.220], 1.0 [X^2^ (2)=3.755; P=0.153], 3.1 [X^2^ (2)=3.755; P=0.153], 6.1 [X^2^ (2)=0.217; P=0.897], 8.8 [X^2^ (2)=0.482; P=0.786], 13.2 [X^2^ (2)=1.427; P=0.490], 15.6 [X^2^ (2)=0.118; P=0.943], and 19.8 cpg [X^2^ (2)=0.891; P=0.641].

**Figure 3:**
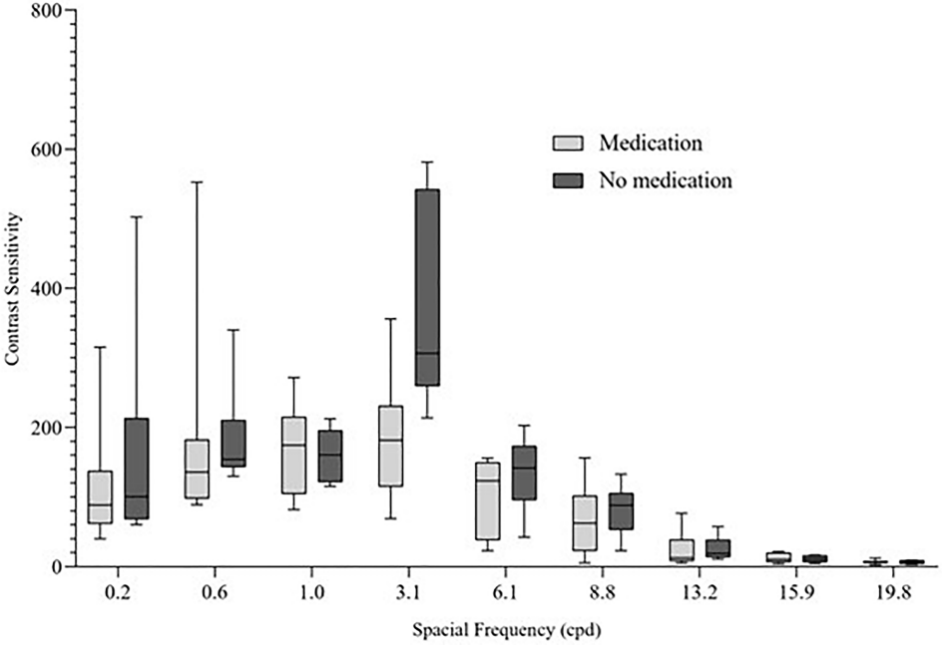
Contrast sensitivity values for COVID-19 subgroups by medication use during the period of symptoms. Lines in boxes are median and interquartile range and whiskers represent maximum and minimum values. cpd: cycles per degree.

SG participants were also subdivided by use of medication during the period of COVID-19 symptoms. Bonferroni correction was applied and all effects were tested at the 0.025 significance level. The Mann-Whitney test showed significant differences regarding the use of medication only for the CSF of frequency 3.1 cpd (U=1.000; P=0.003; r=-0.78). No statistically significant differences were found for spatial frequencies 0.2 (P=0.643), 0.6 (P=0.817), 1.0 (P=0.298), 6.1 (P=0.562), 8.8 (P=0.487), 13.2 (P=0.563), 15.9 (P=0.817), and 19.8 cpg (P=0.355). Figure 3 presents a boxplot with the contrast sensitivity values for the subgroups.

## Discussion

The main objective of this study was to assess whether COVID-19 affected the CSF of individuals exposed to the virus. CSF is a measure that describes the visual system's ability to discriminate objects or spatial frequency patterns at varying contrast levels. The hypothesis posited that exposure to COVID-19 would alter CSF or impair the visual system's ability to discriminate low, medium, and high spatial frequencies. The results showed a reduction in contrast sensitivity only at high spatial frequencies, partially supporting the hypothesis that COVID-19 exposure diminishes contrast sensitivity for sine-wave gratings.

Our results are in contrast with those of another study on the topic, which found no statistically significant differences in contrast sensitivity in participants diagnosed with the disease (22). As reported in the introduction, the effects of COVID-19 on CSF are still unclear and the previous study performed the tests remotely, which can reduce the accuracy of the tests.

Changes in the CSF or in the ability of the visual system to discriminate sinusoidal gratings were expected considering that COVID-19 is a potentially neuroinvasive disease that affects the CNS (16) and that ACE2 and TMPRSS2 receptors are present in ocular tissue (14), especially in the retina (25). Studies have reported that SARS-CoV-2 uses ACE2 and TMPRSS2 as entry receptors into the human body (26) and that cell tropism can occur in the retina after the virus binds to these receptors (11,12).

Furthermore, studies have indicated that COVID-19 can cause changes in the retina (4,6), the structure responsible for processing visual information and establishing fundamental limits on what can be seen (27). Therefore, changes in the retinal circuit, such as those caused by COVID-19, may propagate, generating changes in the retinogeniculostriate pathway and consequently in the visual processing of information or contrast detection. These findings are further supported by animal studies reporting that coronaviruses are known to cause visual manifestations in different species (10), such as retinal degeneration in mice (28) and pyogranulomatous anterior uveitis, choroiditis with retinal detachment, and retinal vasculitis in felines (29). Although the visual implications of the new coronavirus infection in humans have not been widely documented and studies indicate low levels of ocular tissue tropism (9) and viral RNA in the retina (30), animal research has demonstrated that coronaviruses can cause visual manifestations through distinct mechanisms and levels (3).

In principle, however, the reduction in CSF at high spatial frequencies only cannot be explained solely by the presence of ACE2 and TMPRSS2 receptors in the ocular tissue (14), considering that low and medium frequencies are not altered (Figure 2). It is likely that COVID-19 has neuroinvasive potential (16) that affects the behavior of the visual system and perhaps the visual processing of low, medium, and high spatial frequencies in different ways.

High spatial frequencies can change because visual processing of these frequencies is the last to mature, making it the first to be affected by various factors, such as aging and pathologies (21,31). Considering that the average time between COVID-19 diagnosis and study participation was approximately 167 days, this may not have been sufficient time to cause changes in visual processing of low and medium frequencies. Additionally, the impact of COVID-19 on the visual and neural systems may vary depending on factors such as the severity of the infection, the presence of neurological or ophthalmological symptoms, and the individual's overall health (3). As we are dealing with hypotheses about a novel disease, many uncertainties regarding its long-term effects still persist.

The susceptibility of high spatial frequencies to impairments is well documented in various conditions, including viral infections and neuropsychiatric disorders, where fine-detail discrimination is significantly affected (21). These findings suggest that similarities in neural circuits and mechanisms across these conditions can impact multiple systems and contribute to long-term impairments. It is therefore plausible that COVID-19 initially causes deficits in high spatial frequencies, with the potential to affect other frequencies if the infection persists. This implicates further investigation and also serves as a valuable model for understanding the broader influence of COVID-19 on the visual system.

On the other hand, it is too early to say, based on the psychophysical results of the present study, that COVID-19 asymmetrically alters putative brain mechanisms specialized in the processing of spatial frequencies. There are still many gaps regarding the extent of the damage caused by the disease, such as the impact on visual functions (17). However, the literature indicates specific pathways for processing low, medium, and high spatial frequencies, as well as specialization for some visual brain functions (32), including processing of details and high spatial frequencies (33). Psychophysical (33,34) and neuroimaging studies (35) corroborate the hemispheric selectivity for spatial frequency bands.

The type of stimulus used in the study may also signal other implications of COVID-19. The literature indicates that different stimuli are processed by different visual areas (36) and that the Cartesian sinusoidal gratings, as used in this study, are processed in the primary visual cortex (visual area V1) (37), which is responsible for the first level of cortical processing of visual information from the retina. Thus, considering the stimulus used, the alterations found in the CSF may also suggest alterations in the primary visual cortex. Corroborating this idea, a case report demonstrated that damage to the V1 visual area can reduce contrast response functions measured in the injured hemisphere, that is, it can cause impairments in the detection of stimuli at a range of contrast levels (38).

On the other hand, we did not find significant differences between the subgroups regarding the time since diagnosis for any of the spatial frequencies. This may have occurred due to the low number of participants in each subgroup, which may have prevented possible effects from being evidenced in the analysis, and a large variation in data.

Although the results indicated that COVID-19 can cause changes to the visual system, the underlying mechanisms and dimensions are still unknown. Furthermore, can alterations in only high spatial frequencies affect the functional aspects of vision? The literature suggests that the conventional pathway for visual scene processing follows a sequence from coarse aspects (low frequencies) to fine details (high frequencies) (39). In the context of a neuroinvasive pathology, which can affect areas involved in this processing, if no changes are observed in low spatial frequencies, individuals diagnosed with COVID-19 may not immediately notice functional visual changes. This is because the brain can temporarily compensate for the loss of finer details while maintaining overall perception.

In addition, the measure used in this study evaluates the visual pathways that process low, medium, and high spatial frequencies at contrasting photoptic levels. In this sense, even though receptors for SARS-CoV-2 are also present in the retina (13-15), the site of alterations cannot be located by psychophysical studies, but with electrophysiological studies such as electroretinogram and electroencephalography, in addition to neuroimaging studies with optical coherence tomography. Therefore, further studies should be carried out with these techniques to better assess the possible mechanisms and areas of alterations.

This study had some limitations, such as a small sample size, which prevented comparisons with subgroups of individuals who had the asymptomatic form of the disease, mild symptoms, or severe symptoms, since more than 90% of the group consisted of oligosymptomatic patients.

## Conclusions

Our findings indicated that COVID-19 may impair visual processing, particularly by reducing contrast sensitivity at high spatial frequencies. This suggested that COVID-19 could impact visual pathways and spatial frequency processing in a diffuse manner. Furthermore, it was not possible through a psychophysical study to determine whether the impairment in high spatial frequency processing is due to subcortical changes (e.g., in the retina), cortical changes, or along the retinogeniculostriate pathway.

The use of robust psychophysical methods and high-density intra-subject measures supports the reliability of the findings. Future research should expand on this work with larger, more diverse populations, and employ complementary techniques such as electrophysiology and imaging. These approaches will help clarify the pathways and mechanisms involved and may contribute to early diagnostics, targeted interventions, and rehabilitation strategies for patients affected by COVID-19.
